# Supplementary material for: Influence of Sex and Body Size on the Validity of the Microsoft Kinect for Frontal Plane Knee Kinematics During Landings
Source: Sensors (Basel). 2025 Sep 8;25(17):5593. doi: 10.3390/s25175593 (PMC12431473; doi:10.3390/s25175593)
Supplement: Supplementary file 1 [file sensors-25-05593-s001.zip › sensors-3806320-supplementary.pdf]

Supplementary Materials

Table 1. Summary of ANOVA Results.

	Interaction Effects								Main effects					
	Method x Sex x BMI		Method x BMI		Method x Sex		Sex x BMI		Method		Sex		BMI	
	F	*p*	F	*p*	F	*p*	F	*p*	F	*p*	F	*p*	*F*	*p*
KASR at IC	0.12	0.74	10.02	**<0.01**	0.80	0.38	0.68	0.41	155.32	**<0.01**	2.04	0.16	0.02	0.88
KASR at PKF	2.92	0.96	2.32	0.14	0.49	0.49	1.52	0.23	15.17	**<0.01**	2.94	0.10	0.25	0.62
KAA at IC	1.08	0.31	7.17	**0.01**	2.38	0.13	0.73	0.40	5.47	**0.03**	0.14	0.71	18.68	**<0.01**
KAA at PKF	0.22	0.64	12.99	**<0.01**	0.83	0.37	1.33	0.26	25.73	**<0.01**	0.17	0.68	3.95	**0.05**

Notes: Bold denotes significance at P <0.05; KASR: knee–ankle separation ratio; KAA: knee abduction angle; IC: initial contact; PKF: peak knee flexion.

Table S2. Peak Knee Flexion Angles by Sex and BMI.

		**Low BMI**	**High BMI**	**Total**
Peak Knee Flexion Angles (°) (SD)	Female	-90.164 (14.684)	-71.115 (9.976)	-80.138 (15.526)
	Male	-85.356 (12.135)	-74.216 (9.327)	-79.786 (11.984)
	Total	**-87.633 (13.249) ***	**-72.666 (9.533) ***	

Notes: * indicates a significant difference, p<0.001, between measurements. The mean difference for peak knee flexion between the low and high BMI groups is -15.094° (standard error, 3.726; 95% confidence interval, -7.529 to -22.659).

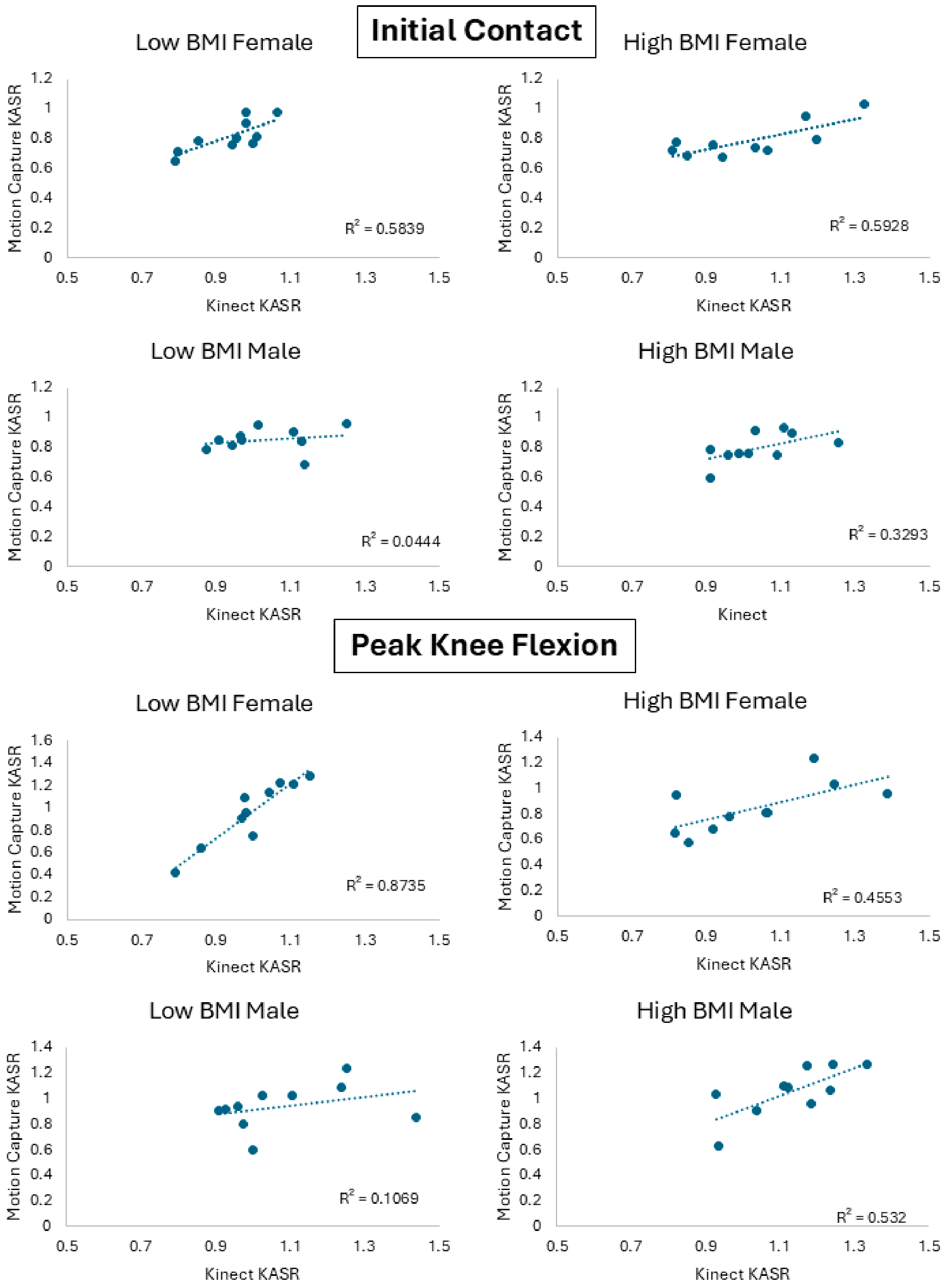

Figure S1. Scatterplots between Kinect and Motion Capture measurements of Knee Ankle Separation Ratio (KASR).

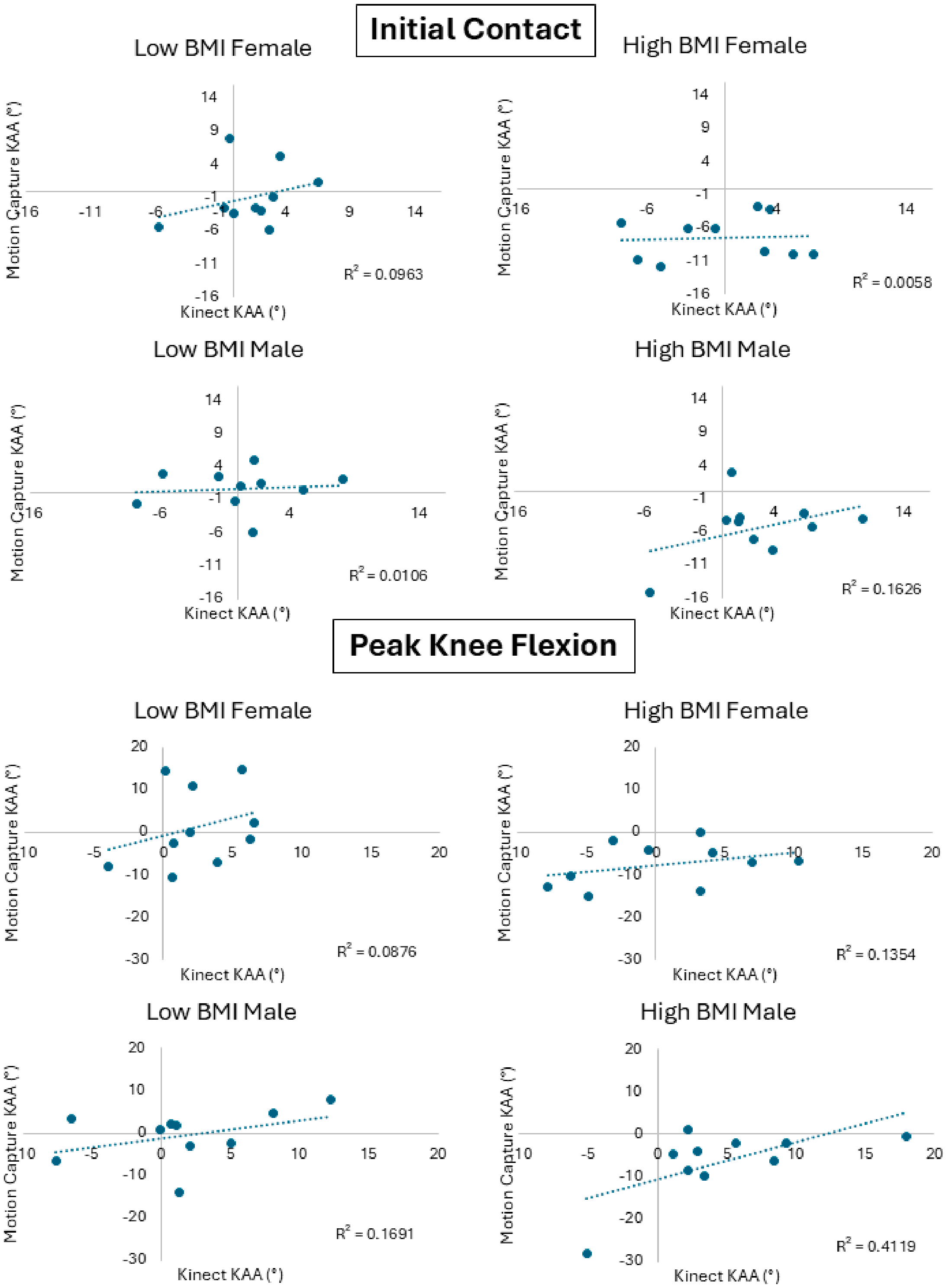

Figure S2. Scatterplots between Kinect and Motion Capture for Knee Abduction Angle (°).
